# The behaviour of giant clams (Bivalvia: Cardiidae: Tridacninae)

**DOI:** 10.1007/s00227-014-2545-0

**Published:** 2014-10-02

**Authors:** Pamela Soo, Peter A. Todd

**Affiliations:** Experimental Marine Ecology Laboratory, Department of Biological Sciences, National University of Singapore, 14 Science Drive 4, Blk S3 02-05, Singapore, 117543 Singapore

## Abstract

Giant clams, the largest living bivalves, live in close association with coral reefs throughout the Indo-Pacific. These iconic invertebrates perform numerous important ecological roles as well as serve as flagship species—drawing attention to the ongoing destruction of coral reefs and their associated biodiversity. To date, no review of giant clams has focussed on their behaviour, yet this component of their autecology is critical to their life history and hence conservation. Almost 100 articles published between 1865 and 2014 include behavioural observations, and these have been collated and synthesised into five sections: spawning, locomotion, feeding, anti-predation, and stress responses. Even though the exact cues for spawning in the wild have yet to be elucidated, giant clams appear to display diel and lunar periodicities in reproduction, and for some species, peak breeding seasons have been established. Perhaps surprisingly, giant clams have considerable mobility, ranging from swimming and gliding as larvae to crawling in juveniles and adults. Chemotaxis and geotaxis have been established, but giant clams are not phototactic. At least one species exhibits clumping behaviour, which may enhance physical stabilisation, facilitate reproduction, or provide protection from predators. Giant clams undergo several shifts in their mode of acquiring nutrition; starting with a lecithotrophic and planktotrophic diet as larvae, switching to pedal feeding after metamorphosis followed by the transition to a dual mode of filter feeding and phototrophy once symbiosis with zooxanthellae (*Symbiodinium* spp.) is established. Because of their shell weight and/or byssal attachment, adult giant clams are unable to escape rapidly from threats using locomotion. Instead, they exhibit a suite of visually mediated anti-predation behaviours that include sudden contraction of the mantle, valve adduction, and squirting of water. Knowledge on the behaviour of giant clams will benefit conservation and restocking efforts and help fine-tune mariculture techniques. Understanding the repertoire of giant clam behaviours will also facilitate the prediction of threshold levels for sustainable exploitation as well as recovery rates of depleted clam populations.

## Introduction

Giant clams (Bivalvia: Cardiidae: Tridacninae) are the largest living bivalves. They live in close association with coral reefs throughout the Indo-Pacific (Lucas 1988) where they are ecologically important as biomass for predators/scavengers and substrates for epibionts, in addition to physically contributing topographic relief (important as nurseries for fish) and calcium carbonate to the reef framework (Govan et al. 1993; Cabaitan et al. 2008; Accordi et al. 2010). Giant clams have also benefitted humans for millennia, through provision of food and materials (Hviding 1993). Unfortunately, harvesting for local consumption (Hester and Jones 1974), export of wild specimens for the aquarium trade (Wabnitz et al. 2003) and habitat degradation (Newman and Gomez 2000) have led to population declines (Alcala 1986; Braley 1987; Tan and Yasin 2003) and extirpations (Neo and Todd 2012a).

Giant clams are marine mollusc equivalents of ‘charismatic megafauna’ that can act as flagship taxa—drawing attention to the ongoing destruction of coral reefs and associated biodiversity. Hence, their conservation and study have a particular importance. Giant clam research has been reviewed a few times, with the primary emphases being their biology, nutrition and mariculture (e.g. Rosewater 1965; Munro and Heslinga 1983; Lucas 1994). No review of giant clams has focussed on their behaviour, yet this component of their autecology is critical to their life history and deserves attention. Here, we seek to address this gap by collating and, where possible, synthesising what is presently known regarding giant clam spawning, locomotion, feeding, anti-predation behaviour and stress responses. The effects of diseases or parasites on behaviour are not examined. We start by providing some phylogenetic and biological background on these iconic invertebrates.

### Tridacninae Lamarck, 1819

Tridacninae currently compromises 12 extant species in two genera: *Tridacna* and *Hippopus*, i.e. *T. gigas* Linnaeus 1758; *T. derasa* Röding 1789; *T. squamosa* Lamarck 1819; *Tridacna noae* Röding 1798 (recently separated from *T. maxima* by Su et al. 2014); *T. maxima* Röding 1789; *T. crocea* Lamarck 1819; *T. mbalavuana* Ladd 1934; *T. squamosina* Sturany 1899 (previously known as *T. costata*); *T. rosewateri* Sirenko and Scarlato 1991; an undescribed cryptic *Tridacna* sp. (Huelsken et al. 2013); *H. hippopus* Linnaeus 1758; and *H. porcellanus* Rosewater 1982. The main differences between the two genera are that *Hippopus* have interlocking teeth at their byssal orifice (Lucas et al. 1991), and they open their valves further apart than *Tridacna* (Lucas 1994), but they do not extend their mantles laterally beyond their valve margins. These highly specialised bivalves are mainly distributed within the tropical Indo-Pacific region although three species, *T. maxima*, *T. squamosa* and *T. squamosina*, are found as far west as east Africa or the Red Sea (Rosewater 1965; Othman et al. 2010). Giant clams are markedly stenothermal and thus restricted to warm waters (Purchon 1977, p. 337). Typically living on sand or attached to coral rock and rubble by byssal threads, they are prominent inhabitants of coral reefs—not only due to their large size, but also because of their intricately patterned and pigmented mantle tissues (Yonge 1975; Calumpong 1992).

Similar to most other bivalves, giant clams are filter feeders. They pump water into their mantle cavity through an inhalant siphon and filter plankton using ciliated tracts on their gills (Hardy and Hardy 1969). The great size exhibited in giant clams cannot be achieved by these ctenidial feeding mechanisms alone (Purchon 1977); rather, it is accomplished together with symbiotic photosynthetic dinoflagellate algae, or zooxanthellae (genus *Symbiodinium*), that live within the mantle tissues (Kawaguti 1950, 1968). Zooxanthellae do not pass from one generation to another (LaBarbera 1975; Jameson 1976) and are permanently established only after metamorphosis from larva to juvenile (Fitt and Trench 1981).

Giant clams are protandrous hermaphrodites (Wada 1952), but otherwise follow the typical bivalve mollusc life cycle (Lucas 1994). Sperm is released, followed by eggs, into the water column where fertilisation takes place. Within a day, the embryo develops into a free-swimming trochophore larvae. Straight-hinge veligers (~160 μm) form by day two and develop a ciliated velum for locomotion and feeding. Transition to the pediveliger stage (~200 μm) over the next few days is marked by the formation of a foot and two-valved shells. During this stage, the pediveliger larvae crawl on the substrate in search of suitable sites for settlement and metamorphose into early juveniles (or spats) within 2 weeks of spawning (Ellis 1998; Blidberg 2004). Giant clams thus spend a short yet critical period of approximately 9 days as pelagic larvae compared to a much longer adult stage. The exact lifespan of tridacnines has not been ascertained, although it is estimated to vary between eight to several hundred years (Comfort 1957; Bonham 1965; Rosewater 1966). Contemporary giant clams do not commonly survive to great ages, possibly due to human activities that continue to threaten population numbers (Yamaguchi 1977; Guest et al. 2008).

Giant clams are distinct from their relatives in the family Cardiidae (cockles) due the unique rearrangement of their internal organs over evolutionary time. Rather than the hinge facing upwards (the normal position for cockles) and the foot protruding downwards, the hinge has rotated around the viscero-pedal mass so that it is on the underside (adjacent to the substrate) and aligned with the foot and byssal gape (Yonge 1975). This means that their enlarged dorsal siphonal mantle, that houses symbiotic zooxanthellae, is directed upwards towards the sunlight (Norton and Jones 1992). Their byssus is orientated downwards and used to attach the clam to hard coral reef substrates. Part of this rotation is reflected in their early ontogeny: although giant clam larvae go through typical bivalve veliger and pediveliger stages, they then undergo a transformation that results in the umbo and hinge positioned alongside the byssal gape (Yonge 1982). Giant clams still bear some similarities to other members of the Cardiidae, for example predator detection via chemoreception (e.g. in the common cockle *Cerastoderma edule*; Romano et al. 2011) and a photosymbiotic relationship (in some fragines, Kirkendale 2009; the heart cockle, Kawaguti 1950; and possibly in the heart/basket cockle *Clinocardium nuttallii*, Hartman and Pratt 1976), but they are behaviourally quite separate in many ways. As Yonge (1982, p. 770) notes: ‘While basic structure, both of mantle/shell and viscero-pedal mass, indicates association with the Cardiidae…, the totally distinctive structure of the Tridacninae indicates long and intimate association with coral reefs’. This shallow, high irradiance habitat of giant clams has had profound effects on the evolution of their behaviour.

Giant clams serve various important roles in the coral reef ecosystem. Their calcium carbonate shells act as nurseries for fish (Cabaitan et al. 2008), as well as providing spatial refuge for smaller filter-feeding epibionts such as barnacles, polychaetes and sponges (Elfwing et al. 2003; Vicentuan-Cabaitan et al. 2014). Through their symbiotic relationship with zooxanthellae, giant clams not only contribute to reef productivity, but indirectly function as natural biofilters of dissolved nutrients (Mingoa-Licuanan and Gomez 2002). Throughout most of their geographical range, giant clams have traditionally been harvested for subsistence (food) and commercial (shell craft products) purposes but, more recently, demand by the aquarium trade has also placed a strain on their numbers (Mingoa-Licuanan and Gomez 2002; Soo et al. 2011). Illegal poaching, habitat degradation and reduced habitat range have substantially depleted giant clam populations, and local extinctions have been reported in the Philippines, Malaysia and Singapore (Alcala et al. 1986; Tan and Yasin 2001a; Guest et al. 2008; Neo and Todd 2012b). In addition, global climate change and associated high sea surface temperature events have led to giant clams ‘bleaching’ (i.e. the breaking down of the symbiotic relationship between the clam and their *Symbiodinium*) (Gomez and Mingoa-Licuanan 1998). Given the deleterious effects of human activities on their numbers worldwide, the trade of most giant clam species is currently regulated under Appendix II of the Convention of the International Trade of Endangered Species of Wild Flora and Fauna (CITES) (http://www.cites.org/). In an effort to conserve populations, many Indo-Pacific countries have restricted harvesting and developed mariculture for commercial purposes and the restocking of depleted reefs. Conservation strategies for these mega-invertebrates have become an important component of numerous coral reef management schemes (e.g. Gomez and Mingoa-Licuanan 2006; Neo and Todd 2012a).

### Giant clam research

Taxonomic descriptions of Tridacninae date back to 1758, but giant clam biology more generally escaped the attention of scientific research until the mid-nineteenth century (e.g. Vaillant 1865), perhaps surprising considering the evolutionary significance of the clam-zooxanthellae symbiosis (Brock 1888). In a bibliography of giant clam literature (Munro and Nash 1985), more than 70 % of the scientific papers compiled were published after 1970. This surge in research interest started when biologists first successfully cultured giant clam larvae through to metamorphosis (e.g. LaBarbera 1975; Jameson 1976). Numerous technical challenges had to be surmounted, including the unpredictability of spawning behaviour, unascertained dietary requirements in early life leading to high mortality rates, and difficulty in handling small metamorphosed juveniles (Yamaguchi 1977). There is now, however, a substantial body of data available on symbiosis and nutrition (e.g. Fitt and Trench 1981; Trench et al. 1981), reproduction (e.g. Gwyther and Munro 1981; Neo et al. 2011), shell morphology (e.g. Chan et al. 2009; Neo and Todd 2011a), and growth (e.g. Munro and Gwyther 1981; Guest et al. 2008). Field research has mainly concentrated on *T. gigas*, the largest and fastest growing species, and *T. maxima*, which has the most widespread distribution (Adams et al. 1988). Even though the biology, exploitation and mariculture of giant clams have been well-studied (reviews in Munro 1993; Lucas 1994; Hart et al. 1998), their ecology and behaviour are relatively poorly known.

One of the earliest articles on giant clams to contain behaviour-related information was published in 1865 (Vaillant 1865). It notes that these unique bivalves are not sessile and describes the relationship between shell weight and clam mobility as development progresses. Our search of the literature published between 1865 and 2013 yielded approximately 100 articles that included behavioural information. Nearly a third focus on spawning patterns and only a handful explore other behaviours quantitatively (e.g. Suzuki 1998; Huang et al. 2007). Brief qualitative or anecdotal notes (e.g. regarding larval studies in LaBarbera 1975; Jameson 1976; Fitt and Trench 1981) are much more common.

## Spawning

Early research determined that tridacnines are functional protandric hermaphrodites that spawn sperm first and then eggs (Wada 1952, 1954). Such reproductive behaviour is not known to occur in other hermaphroditic bivalves (including the great majority of cockles), which generally discharge both sperm and eggs simultaneously. In general, male gonads mature at 2–3 years, whereas female gonads mature at 3–4 years (Lucas 1994). Larger species such as *T. gigas*, however, take longer to become functionally reproductive (e.g. 10 years) (Gomez et al. 2000).

Wada (1954) first described spawning in adults occurring in three distinct phases: the discharge of gametes from gonads, rhythmic contraction and relaxation of adductor muscles, and mantle movements (Lucas 1988; Husin et al. 2001; Tan and Yasin 2001b). Both sperm and egg spawning behaviours are similar (Wada 1954). Several rhythmical valve contractions are usually observed prior to either event, but the latent periods between sperm and egg release appear to show interspecies variation (Alcazar 1988). The simultaneous discharge of both sperm and eggs is abnormal, which Wada (1954) highlighted as indicative of an unhealthy adult. The fecundity of giant clams is exceptionally high (Yamaguchi 1977), even described as the ‘*pinnacle of fecundity in the animal kingdom*’ (Lucas 1994, p. 187). The number of oocytes released per spawning adult (in the millions) is related to clam size (Alcazar and Solis 1986; Alcazar 1988; Husin et al. 2001). The period for fertilisation is limited by the short viability span of gametes, reported to be 4–6 h after release (Tan and Yasin 2001a). During ex situ fertilisation for mariculture, sperm and eggs are mixed typically within 15 min of egg release (Ellis 1998).

### Reproductive seasonality and spawning cues

As with other cardiids (e.g. Galluccil and Galluccil 1982), giant clams display seasonal synchronous spawning within metapopulations (Heslinga et al. 1984; Shelley and Southgate 1988). Egg spawning of one individual will chemically trigger sperm spawning of nearby clams, thus ensuring gametes meet (Lucas 1988; Tan and Yasin 2001a). Adults are relatively sedentary, and given their reliance on external fertilisation, reproductive success is related to the density of the population (Adams et al. 1988; Downing et al. 1993). Individuals in close proximity tend to be more reproductively synchronised compared to those more spatially separated (Braley 1986).

Interspecific variation in reproductive seasonality is widespread in giant clams. The period for gamete maturation varies across species, but can be up to 4 months duration (Tan and Yasin 2001a). The majority of studies to date have been conducted over too brief a period for peaks in spawning intensity to be clearly defined (Munro and Heslinga 1983); however, some giant clams exhibit diel and lunar patterns, and seasonality is likely to be influenced by latitude and/or geographical locality (Braley 1984; Heslinga et al. 1990; Tan and Yasin 2001a) even at relatively small scales. For instance, populations of *T. squamosa* within the Tioman Archipelago, Malaysia, do not share the same spawning season (e.g. Pulau Tioman: September–April; Pulau Pemanggil: May–November) (Tan and Yasin 2001a).

Despite considerable research, no consensus has been reached on spawning frequency or natural mass spawning cues (Svane 1996). Temperature (Stephenson 1934) and water movement (Jameson 1976) have been suggested as stimuli for spontaneous spawning, but these have been dismissed by other researchers (e.g. LaBarbera 1975; Jameson 1976; Beckvar 1981). Compared to sperm release, observations of in situ egg spawning events are very rare even when gonads are ‘ripe’ (Braley 1984, 1986; Lucas 1994). Specific environmental cues, such as phytoplankton blooms and temperature rises, may only occur in certain years and can account for sporadic egg spawning (Braley 1986; Tan and Yasin 1998). It is also possible that more than one cue is required to trigger synchronous spawning (Braley 1986); however, the potential synergistic effect of multiple environmental parameters are yet to be investigated (Tan and Yasin 1998).

Giant clams have been induced to spawn in laboratory conditions using cues such as macerated or freeze-dried gonads (Jameson 1976; Gwyther and Munro 1981), intra-gonadal injections of serotonin (Braley 1985; Crawford et al. 1986) and stripped eggs, either fresh or held overnight at 8 °C (LaBarbera 1975). The effectiveness of gonad suspension as a spawning stimulus is not only species-specific but also dependent on the sexual maturity, or ripeness, of the gametes (Wada 1954). Munro et al. (1983) postulated that the active cue is carried by the eggs and not the spermatozoa. The neurotransmitter serotonin can initiate spawning behaviour within five to ten minutes after injection into the gonads (Braley 1986; Husin et al. 2001). Braley (1986) mentioned thermal stress as a trigger for spawning in tank-held *T. gigas* and *T. derasa* but noted that the broodstock’s physical and reproductive condition may be lowered.

## Locomotion

Many benthic marine invertebrates possess complex life cycles characterised by planktonic larval phases followed by bottom-dwelling juvenile and adult stages (Thorson 1950; Watzin 1986). As larvae, dispersal by currents and searching for optimal settlement sites are key factors of early-life survival and therefore have significant effects at the individual, population as well as community levels (Rodríguez et al. 1993; Tan and Yasin 2001a). Starting with settlement and metamorphosis, locomotive activity declines with age, reflecting both anatomical constraints, in terms of byssus atrophy and shell weight-to-body mass ratio (Heslinga 1989), as well as ecological needs—where more energy is channelled into growth and reproduction.

### Larvae

Within 7–16 h after fertilisation, the first larval stage to exhibit controlled movement is the ciliated gastrula (Table 1). LaBarbera (1974) and Jameson (1976) recorded active rotation within the water column by the gastrulae of numerous species, while those of *H. hippopus* remained on the bottom of the rearing tank (Fitt et al. 1984). Trochophores develop by 24 h post-fertilisation (Jameson 1976; Tan and Yasin 2001a) and are free-swimming and active (Fitt et al. 1984; Alcazar and Solis 1986). Cone-shaped *T. squamosa* trochophores have been observed swimming in a spiralling motion (LaBarbera 1974), and such locomotion may be facilitated by the presence of apical flagella on the anterior end (Raven 1966; Carriker 1990). Trochophore larvae do not orientate towards light (Fitt et al. 1984).

**Table 1:** Locomotive behaviour exhibited by giant clam larvae

Developmental stage	Time after fertilisation	Locomotive behaviour	Species
Ciliated gastrula	7–16 h	Active rotation, remains at bottom	TC, TM, TS, HH
Trochophore	12–24 h	Free-swimming, active at surface, translation parallel to and rotation around long axis	TC, TG, TM, TS, HH
Veliger	18–72 h	Active swimming throughout medium	TC, TG, TM, TS, HH, HP
Pediveliger	4–19 days	Alternately crawling, swimming and gliding off bottom	TC, TM, TS, HH, HP

TC = *Tridacna crocea*, TG = *T. gigas*, TM = *T. maxima*, TS = *T. squamosa*, HH = *Hippopus hippopus*, HP = *H. porcellanus*

**Fig. 1:**
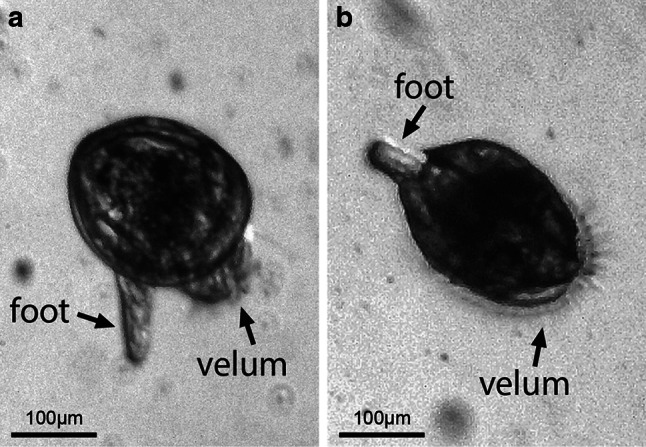
Swimming in 8-day-old *T. squamosa* pediveligers: **a** a pediveliger just about to leave the substrate, **b** a pediveliger swimming using its velum—possibly using its foot as a stabiliser

The transition to veliger is characterised by the development of the velum—which occupies approximately two-thirds the volume of the shell cavity (Carriker 1990; Ellis 1998). Swimming is one of the functions of the velum (along with respiration and food collection), and it persists through to the pediveliger stage but degenerates subsequently (i.e. by juvenile stage). While the densely ciliated velum creates spiral swimming movements, the extension of the foot could possibly serve as a stabiliser (Fig. 1). The simultaneous use of the velum and foot has not been formally described but has been observed in *T. squamosa* (Mei Lin Neo, personal communication). Giant clam veligers have species-specific arrangements of the muscular system, which comprises of velar retractors for swimming and adductor muscles for valve closure (Bayne 1971a; Hickman and Gruffydd 1971; Jameson 1976).

The transition from the veliger to pediveliger stage is critical as it represents free-swimming larvae leaving the water column to metamorphose into benthic juveniles (Carriker 1990). In giant clams, pediveligers generally develop 6–14 days after fertilisation. Two anatomical features distinctive of this stage are a two-valved shell, and a ciliated foot with a sensitive propodial tip for crawling (Carriker 1990; Alcazar et al. 1987). The presence of a functionally active foot signifies larval competency for benthic locomotive activities and active exploration for suitable substrates (Purchon 1977).

The development of the posterior adductor muscles coincides with the time of visible pediveliger locomotion (Jameson 1976). Other structures that develop at this stage are the byssus complex and statocysts, and overall these changes give rise to a suite of behaviours observed during the pediveliger stage, including crawling, gliding, swimming, and substrate testing and orientation. *Tridacna squamosa* pediveligers were observed crawling 9 days after fertilisation by LaBarbera (1975). Crawling involves anchoring the tip of the foot to the substrate, followed by the retraction of the pedal muscles to pull the body forwards (Jameson 1976). Gliding movement, i.e. a continuous ‘pull’, is created by the ciliated tip of the extended foot (Jameson 1976). This behaviour has been exhibited by the pediveligers of *T. crocea*, *T. maxima* and *H. hippopus* but not in *T. squamosa*, even though the surface of the foot in this species is ciliated (LaBarbera 1975).

#### Substrate testing and orientation

Giant clam pediveligers are believed to possess sensory and effector mechanisms within the foot similar to those in the blue mussel *Mytilus edulis* and giant scallop *Placopecten magellanicus* (Culliney 1974; Lane and Nott 1975). The foot is covered in cilia (at least for *T. crocea, T. maxima, T. squamosa* and *H. hippopus*) and likely functions as a sensory organ—testing the surrounding substrate and orienting the clam (Jameson 1976). Neo et al. (2009) reported that, in *T. squamosa* larvae, the tip of the extended foot moved in a sinoidal fashion and appeared to sense the substrate prior to crawling. Similar searching behaviour has also been observed in the bivalves *Crassostrea virginica*, *Mercenaria mercenaria* and *Ostrea edulis* (Yonge 1960; Cranfield 1973).

Over time, the pediveliger becomes increasingly sedentary due to the increase in shell weight and reduction in velum size. The degeneration of the velum occurs simultaneously with the development of functional gills and usually confines the clam to the substrate it has chosen for settlement (Jameson 1976). In the case where suitable substrates are unavailable, the pediveliger stage may be extended, as in other bivalves such as *Mytilus edulis* and *Placopecten magellanicus* (Loosanoff and Davis 1963; Bayne 1965). Giant clam larvae tend to settle on substrates which offer shelter in the form of grooves and crevices, a behaviour frequently reported in corals (e.g. Petersen et al. 2005a, b). In aquaria, *T. maxima* larvae move towards, and byssally attach to, corners of tanks and the edges of plastic panels (Gwyther and Munro 1981). High substrate rugosity such as coral rubble and rough cement tiles also favours larval settlement (Alcazar and Solis 1986; Alcala et al. 1986; Neo et al. 2009). Calumpong et al. (2003) tested the settlement response of 13-day-old *T. squamosa* larvae on a variety of eight natural and artificial substrates: black pebbles, cement, coral rubble, dead coral, live coral, rough Mactan stone, smooth Mactan stone and *Tridacna* shells. Survival was consistently and significantly highest on rough and smooth Mactan stones, while shell growth was highest on cement.

Giant clam larval settlement can be elicited by chemicals associated with crustose coralline algae (CCA) (Courtois de Vicose 2000; Dumas et al. 2014). CCA is a well-established inducer of settlement and metamorphosis in many reef invertebrate larvae and, in an experiment to test its effect on giant clams, Neo et al. (2009) determined that significantly more *T. squamosa* larvae were attracted to small concrete tiles enriched with CCA covered coral rubble (CCACR) compared to control tiles. Pediveligers appear to be able to sense allelopathic compounds in scleractinian corals (e.g. review by Lang and Chornesky 1990) as they display anti-settling behaviour to live coral *Porites* spp. (Calumpong et al. 2003). Recently, Dumas et al. (2014) demonstrated that *T. maxima* larvae are also attracted to chemicals released into the water by juvenile conspecifics. Together, these results suggest a relatively well-developed chemosensory system in giant clam larvae.

### Juveniles

It is often assumed that after settlement on a suitable substrate, giant clam pediveligers metamorphose into juveniles and lose the ability to move (Calumpong et al. 2003). This notion that juveniles are immobile and permanently attached to the substrate is not valid as they can continue to exhibit two types of movement: rotation and translation (Huang et al. 2007). Rotation is defined as a change of orientation without the approximate centre of the clam being shifted from its initial position, while translation refers to the lateral movement of the clam away from its original position. Huang et al. (2007, page 273) describes *T. squamosa* translation as ‘the foot protruding out of the shell through the byssal orifice to contact the substrate for movement’. How much translation occurs can vary among species and substrates. For example, Toonen et al. (2012) grew four species (*T. squamosa*, *T. maxima*, *T. derasa* and *T. crocea*) on 4-cm-diameter concrete ‘plugs’, but found that all of the *T. squamosa* had moved off these substrates by the end of their experiment. Rapid contraction of the valves also generates a force that supplements locomotion (Stasek 1962; Huang et al. 2007), but leaping of the type described in the cockles *Laevicardium crassum* and *Cardium echinatum* by Ansell (1967), where the foot is used to spring the animal up in the water, has not been recorded. Small individuals of *T. crocea* and *T. squamosa* (10–22 mm) are able to climb vertical surfaces with the aid of byssal threads (Yonge 1936; Huang et al. 2007).

Suzuki (1998) showed that *T. crocea* exhibited more locomotion at night, potentially an adaptation to reduce the risk of visual predation (Suzuki 1998). Soo and Todd (2012) examined the changes in locomotive activity under natural light conditions for *T. squamosa*. Eight individual juveniles were placed in the centre of separate tanks and filmed for 24 h. Locomotion (facilitated by pedal locomotion and valve closure) occurred in four of the eight clams, and only at night. Three of the clams travelled more than 100 mm. Vertical climbing was observed for a 17-mm clam which crawled at least 70 mm up the tank wall in the night and remained there during the daytime. This nocturnal activity appears to be more pronounced in giant clams than in other cardioids such as *Cerastoderma edule* (Richardson et al. 1993).

Existing knowledge of the tactic response of giant clams to directional abiotic stimuli (i.e. light, chemicals, and gravity) is limited to juveniles. Given the tendency for photosynthetic zooxanthellae to move towards illuminated sources (Hollingsworth et al. 2005) and induce a similar response movement in hosts such as coral planula and sea anemones (Atoda 1953; Zahl and McLaughlin 1959), giant clams might also be expected to be phototactic. However, Huang et al. (2007) rejected the hypothesis that *T. squamosa* juveniles move towards light as they found no significant relationship between direction of light source and direction of movement under experimental conditions.

**Fig. 2:**
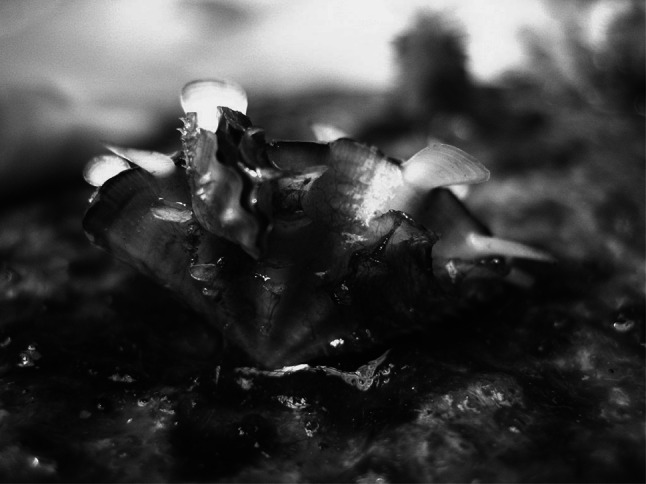
An example of a smaller *T. squamosa* juvenile (8.5 mm) attached to the valve of a larger *T. squamosa* individual (27.8 mm), possibly due to overcrowded conditions

Even though chemical attraction is one of the more challenging biological phenomena to quantify, Huang et al. (2007) demonstrated positive chemotaxis in giant clam juveniles. In a choice experiment using bidirectional water inflow, *T. squamosa* juveniles moved towards the effluent of conspecifics as opposed to clean seawater. Similar results have been found for *T. maxima* juveniles (Dumas et al. 2014). Huang et al. (2007) suggested chemical signalling was a proximate mechanism for non-random aggregation (i.e. clumping), the benefits of which they suggested to be: lower individual risk of predation (e.g. Reimer and Tedengren 1997; Krause and Ruxton 2002), high reproductive success during mass spawning (Adams et al. 1988; Downing et al. 1993) and physical stabilisation against abiotic stresses (Seed 1969; Bertness and Grosholz 1985). By moving non-randomly towards conspecifics while they are small in size, juveniles can form clumps before shell growth and weight become limiting factors to locomotion. A potential negative outcome of clumping is overcrowding and may explain why sometimes smaller individuals climb and attach to the valve surfaces of larger conspecifics (Fig. 2).

**Fig. 3:**
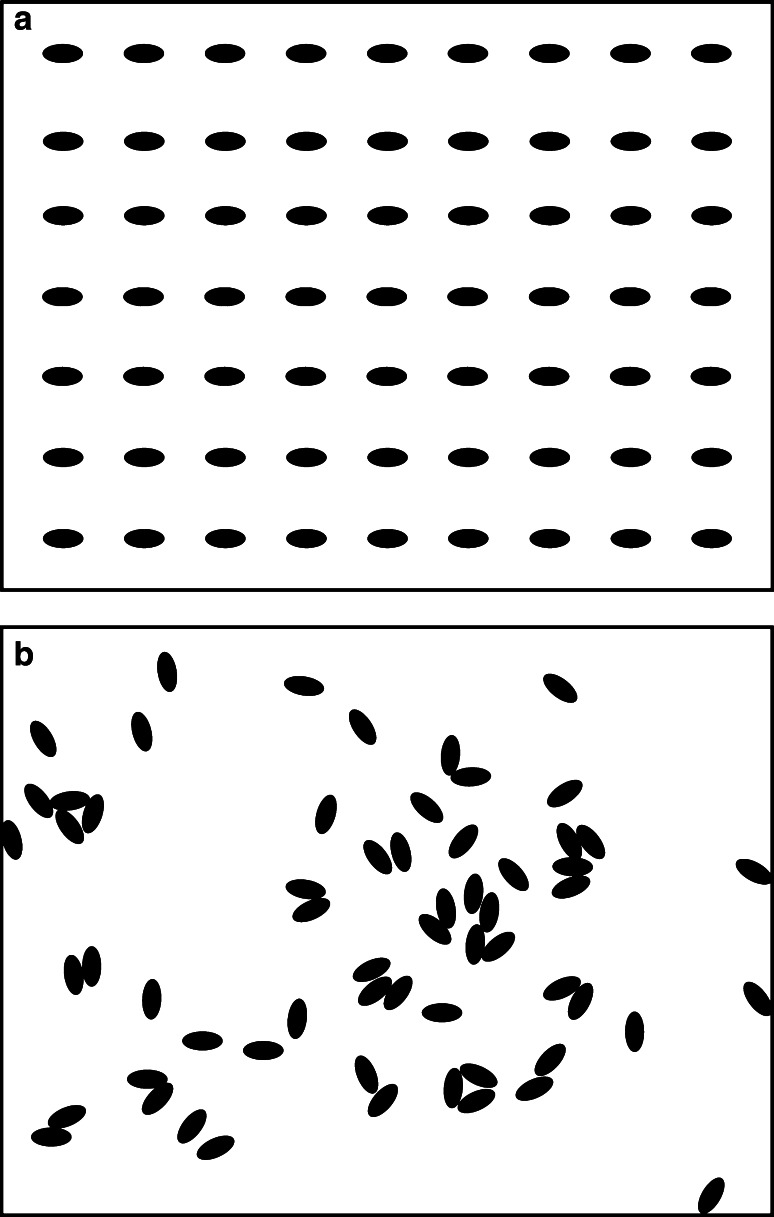
Positions of *T. squamosa* juveniles at the **a** start of the aggregation experiment and **b** after 24 h. Image is based on the video stills in Soo and Todd (2012)

When testing for clumping in juvenile *T. squamosa*, Huang et al. (2007) only recorded positions at the beginning of their experiment and then again at its end (i.e. after 3 days). To gain a better understanding of the locomotive behaviour involved, Soo and Todd (2012) used time-lapse photography to track the aggregation process in 63 *T. squamosa* juveniles. The clams clumped within 24 h (Fig. 3) with mean distance travelled estimated at 280 mm (±SE 34.9). Locomotion away from the initial position was observed in 81 % of the clams, and, in concurrence with the studies on day-night locomotive activity (see above), this was limited to night time (between 8.30 pm and 5.00 am).

Juvenile *T. crocea* and *T. squamosa* frequently climb the vertical surfaces of experimental tanks (Yonge 1936; Huang et al. 2007; Soo and Todd 2012), and Jameson (1976) considered this behaviour to be anti-predatory. Casual observations of *T. squamosa* vertically climbing when exposed to predator effluent (as compared to no-effluent controls) were made by Neo and Todd (2011a), seem to support Jameson’s (1976) hypothesis. However, Soo and Todd (2012) identified geotaxis in the opposite direction in juvenile *T. squamosa* when they showed that significantly more clams (10 out of 16) moved down concrete tiles tilted at a 60° angle than up them. The coral reef is a spatially complex habitat which can provide refuge and settlement areas for many benthic invertebrate taxa (Idjadi and Edmunds 2006); therefore, the ability to move over, climb and descend within this environment is probably highly adaptive.

### Adults

Locomotion (displacement of ~20 cm in 3 days) in a 313-mm shell length *T. squamosa* was observed by Huang et al. (2007), and adults are generally able to right themselves (Fankboner 1971). Studies on tactic responses in mature giant clams are absent.

## Feeding

Fankboner and Reid (1990) noted that tridacnines are not only the largest bivalve to have lived, but also possibly the most opportunistic. Their unique repertoire of feeding modes allows them to adapt to changes in local environment factors (Reid et al. 1984). Starting with a lecithotrophic and planktotrophic diet during larval stages, giant clams then switch to pedal feeding after metamorphosis and this is followed by the transition to a dual mode of filter feeding and phototrophy upon establishing symbiosis with zooxanthellae (Table 2). The reorganisation of feeding at metamorphosis is likely one of the most extreme within the catalogue of giant clam behaviours and probably accounts for the high mortality rates observed during the transition from trochophore to veliger and from pediveliger to juvenile (Fitt et al. 1984).

**Table 2:** Ontological changes in acquiring nutrition

Developmental stage	Diet	Start (duration)	Source/mode of acquiring nutrition
Larva	Lecithotrophic Planktotrophic	Embryo (unknown) Veliger stage	Egg yolk reserves (Fitt et al. 1984) Velum is used for feeding (Carriker 1990)
Pediveligers	Particulate	After metamorphosis (~1 week)	Pedal feeding (Reid et al. 1992): 1. Locomotory pedal feeding 2. Pedal sweep-feeding 3. Pedal probe-feeding
Juvenile, Adult	Heterotrophic Phototrophic	Maturation of ctenidia Establishment of symbiosis	Filter feeding (e.g. Yonge 1936) Photosynthates from symbiotic zooxanthellae (Streamer et al. 1988)

### Larvae

The ability to feed is absent in the early stages of giant clam larval development as they initially rely on nutrients stored in the egg yolk (Heslinga et al. 1990). The formation of a functional filter-feeding apparatus (velum) at the veliger stage suggests a switch to exogenous sources. Within 2–3 days after fertilisation, veligers develop hollow intestines and are demonstrable planktotrophs. They actively uptake flagellates (~5 µm diameter), zooxanthellae and dissolved organic nutrients from the seawater via the mouth (Fitt et al. 1984; Braley 1986; Alcazar et al. 1987). The food is kept in motion by stomach cilia (Jameson 1976).

While growth rates during early life are greatest in veligers, mortality plagues the trochophore–veliger transition (Fitt et al. 1984). Braley (1986) recommended targeting the change from lecithotrophic to planktotrophic feeding to reduce larval death in aquaria. The provision of a mixed micro-algal diet with high lipid composition and fat-soluble nutrients within this time frame is believed to increase the chances of surviving the transition from trochophore to veliger (Fitt et al. 1984; Neo et al. 2013a). Compared with adult clams, symbiotic zooxanthellae are not a major source of nutrition during the larval stages (Fitt et al. 1984). Veliger larvae of *T. gigas* and *H. hippopus* can metamorphose into juveniles in the absence of symbiotic algae and phytoplanktonic food (Gwyther and Munro 1981). An externally introduced food supply, however, appears to increase veliger growth rates as well as shorten the time to metamorphosis (Fitt et al. 1984).

Pedal feeding generally refers to using the foot as a feeding organ to collect food particles on the substrate surface, and this can sustain pediveligers and young juveniles until the inhalant siphon and ctenidial food grooves are functional for filter feeding (Reid and King 1988). *Tridacna gigas* juveniles (and probably those of all giant clam species) possess the behavioural and neurophysiological capacity for three putative forms of pedal feeding (Reid et al. 1992). Forward locomotion over a hard substrate creates an anterior current flow through the pedal gape, allowing small clumps of detritus on the substrate surface to enter the mantle cavity. The second form is anteroposterior pedal sweep-feeding on a sandy substrate, during which the foot extends posteriorly and is swept forward with slight rotation before retracting. Mucus strings produced by the foot traps sediments which are then ingested. Thirdly, while in an upright position, probing of the foot down into the substratum may enable the pedal cilia to gather particulate food. It has yet to be determined how important pedal feeding is during the metamorphosis-ctenidial maturation time gap. The difficulty in quantifying the nutritional significance of this feeding mode is compounded by the establishment of endosymbiotic zooxanthellae during the same period.

Giant clams derive a substantial portion of their food from their symbiotic dinoflagellate algal symbionts (*Symbiodinium*) harboured in their hypertrophied mantle tissues (Lucas, 1994). Species can host multiple clades, for instance DeBoer et al. (2012) found 11 *Symbiodinium* types belonging to clades A, C, and D in *T. crocea* (more clams with C, less with D), *T. squamosa* (more clams with D, less with C) and *T. maxima* (no clear pattern) in Indonesia. They also found that 42 % of the clams sampled simultaneously hosted more than one clade (DeBoer et al. 2012). Research on the early development of giant clams (e.g. LaBarbera 1974) concluded that zooxanthellae are not inherited by the offspring but rather that each generation of giant clams is independently infected with algal symbionts (Stephenson 1934; LaBarbera 1975; Jameson 1976). Hence, larval or juvenile clams must actively acquire zooxanthellae from the environment through feeding. Symbiosis was thought to be only established fully only after metamorphosis (Fitt and Trench 1981; Fitt et al. 1984), when tubules extend from the digestive gland into the developing siphonal tissues. More recently, however, Mies et al. (2012) found greater growth and survival rates in larvae with zooxanthellae, suggesting that the larvae are gaining some benefit from the association at this early stage.

As with the Cardiidae in general, giant clams are opportunistic filter feeders (Yonge 1936; Malham et al. 2012). Details of their ciliary feeding mechanism have been described by Purchon (1955a, 1977) and Morton (1967). The transition from pedal feeding to filter feeding can only take place when the ctenidia has reflexed and formed food grooves (Yonge 1974; Reid et al. 1992). When giant clams filter feed, the valves gape widely and mantle lobes become fully exposed (Wada 1954). Large volumes of seawater, containing planktonic larvae, zooxanthellae, protozoans and particulate matter in mucus flocs released by corals, are pumped through the mantle cavity—especially during daytime (Yonge 1936). The ciliary tracts on the gill remove this material, and water is passed back out through the exhalant siphon (Hardy and Hardy 1969). Giant clams are efficient utilisers of particulate organic matter, retaining on average three quarters of particles 2–50 µm in size. Under turbid water conditions and corresponding reduced light intensity, *T. squamosa* are able to increase filter-feeding rates (Tedengren et al. 2000), a behaviour not observed in other giant clam species (Klumpp and Lucas 1994).

### Juveniles and adults

Both late juveniles and adults share the same feeding behaviours. They obtain nourishment in four ways: (1) autotrophic feeding via the transfer of photosynthates produced by zooxanthellae in the mantle tissues, (2) digestion of zooxanthellae, (3) filter-feeding and (4) uptake of dissolved organic and inorganic molecules (see reviews in Fankboner and Reid 1990; Fitt 1993; Lucas 1994). Three behaviours related to valve movements, waste removal, behaviour during emersion and circadian rhythm, are highlighted below. They are intrinsically linked to both autotrophic feeding and filter feeding, as the efficiency of the former relies on light capture while the latter depends on the rate of water current flowing through the siphons.

Periodically, bivalves rapidly clap their shell valves to expel waste such as foreign matter or pseudofaeces that have entered the infra-branchial cavity. During this process, water can escape from the proximal oral grooves into the infra-branchial cavity and therefore flush out the mucous food cords. Various adaptations to enclose the proximal oral grooves and protect the food train from dislodgement by such violent water movements have been adopted by numerous monomyarian bivalve species, including *Ostrea edulis* and *Pecten maximus* (Gilmour 1964). Interestingly, these structures remain unspecialised in *Tridacna* spp. (Purchon 1977).

Undisturbed giant clams typically have valves that gape open (Yonge 1936; Hickman and Gruffydd 1971; Mingoa-Licuanan and Lucas 1995) and even when intertidal species such as *T. crocea* and *T. squamosa* are exposed by low tides, they continue to display valve gaping (Rosewater 1965; Wilkens 1984; Mingoa-Licuanan and Lucas 1995). Gaping behaviour during emersion is indicative of both active ventilation, as shown in mussels (Bayne 1971b), and aerial photosynthesis—even though the mantle tissues are withdrawn (Lucas et al. 1989). Physiological studies on the effects of emersion on giant clam nutrition have demonstrated that they can photosynthesise out of water (Mingoa-Licuanan and Lucas 1995) and aerial phototrophy in *T. gigas* juveniles is estimated to satisfy more than 100 % of the carbon requirements for metabolism. Thus, the ability of giant clams to maintain energy input through zooxanthellar photosynthesis distinguishes them from other non-photosynthesising intertidal bivalves which also gape when exposed by low tides.

The feeding behaviour in giant clams has a marked circadian rhythm (Fankboner 1971; Morton 1978; Reid et al. 1984; Fankboner and Reid 1990); solar and lunar cycles which determine food availability in turn influence the diurnal feeding activity. At night, they withdraw their mantles and close their valves either half-way or fully, remaining quiescent till dawn (Gwyther and Munro 1981; Heslinga et al. 1984). During this period, weak siphonal activity indicates little respiratory exchange. There is also a lack of response to light, and tactile stimulation elicits only small, sluggish valve adduction (Stasek 1965; Fankboner 1981). 1–3 h prior to sunrise, the response to shadows, water turbulence and tactile stimuli is typical of those during full daylight, i.e. rapid, multiple adductions with expulsions of large volumes of water (Fankboner and Reid 1990).

## Anti-predator behaviour

Natural predators of giant clams vary in their attack mode according to the age or size class of their prey. Young clams are highly vulnerable to crabs (e.g. *Thalamita* spp., *Demania* spp.) that use their chelae to crush the shell valves; wrasses (*Halichoeres* spp.) feed on the byssus and foot of unanchored clams; and pyramidellid and ranellid snails are parasitic (Alcazar 1986). Tooth marks on the outer shell surfaces are indicative of attacks by grazing reef fish (Stasek 1965). For older clams, potential predators include eagle rays, turtles and large benthivorous fish (Bustard 1972; Govan et al. 1993), but their impact is reduced as the clams grow towards escape size (Adams et al. 1988).

Constitutive anti-predatory defences present in giant clams include camouflaging mantle colours and polymorphism (Todd et al. 2009) thick and heavy shells (Lin et al. 2006; Neo and Todd 2011a) and, in some species, sharp shell projections called ‘scutes’ (Ling et al. 2008; Chan et al. 2009). Behavioural defence mechanisms revolve primarily around the closing of valves, mediated by the contraction of adductor muscles (Morton 1967). This shell-closing reaction has been observed at all developmental stages. Mechanical disturbances within a clam’s immediate environment may be interpreted as predatory attacks and elicit various degrees of valve closure, such as rapid muscular adductions or an extended period of remaining shut. The water residing in the mantle cavity is expelled either through the inhalant aperture or exhalant siphon as a jet (Morton 1967). Depending on the strength of the muscular contraction, the valve closure response can function defensively in at least three ways: (1) by reducing the amount of soft tissue exposed and thus vulnerable to attack, (2) by providing locomotion and escape during larval and juvenile stages (e.g. Ansell 1967; Jameson 1976; Huang et al. 2007) and 3) by producing jets of water that can startle fish (Stasek 1965).

### Larvae

When disturbed, giant clam veligers display a fright reaction, i.e. withdrawal of the velum into the shell, closure of valves and passive sinking (LaBarbera 1974), and this represents the only defence mechanism known at this life-stage. It can be observed within 38 h of fertilisation, usually at least 2 h after the calcification of shell rim and the full formation of the retractor and adductor muscles. During subsequent larval stages, the pattern of calcification appears to rapidly reinforce those areas which are necessary to effect the fright reaction and which are subjected to the greatest stress during valve closure. The speed and coordination involved in valve closure may reflect the neuronal innervations of these muscles and their ability to respond to mechanical stimulation (Marois and Carew 1990).

### Juveniles

Giant clams exhibit two forms of visually mediated behaviours: shadow response and ‘sight reaction’ (Stasek 1965; McMichael 1974). The former is characterised by a withdrawal reflex common among bivalves experiencing passing shadows (Land 1968), while the latter also involves mantle withdrawal but this time in response to moving objects even when their shadows do not fall directly on the clam. Several authors have reported dependence on a visual system during daylight hours to avoid attack by reef fish and other predators (Stasek 1965; Wilkens 1986). Giant clam pin-hole eyes can be found along the mantle margins and Land (2003) noted several hundred in *T. maxima* juveniles (150 mm shell length). They constitute a mass of retinal cells devoid of a lens, but the cooperative functioning of small numbers of siphonal eyes could confer directional sensitivity (Fankboner 1981).

During daytime, shadows with abrupt changes in intensity or movement elicit rapid retractions of the mantle coupled with valve adduction, producing a spout of water from the exhalant siphon (Wilkens 1986). This behaviour is thought to be centrally coordinated and serves to startle potential predators via movement of the colourful mantle coupled with the squirting of water (Wilkens 1981). *Tridacna maxima* and *T. squamosa* are more sensitive to shadows and tactile stimuli than *T. gigas* (Fankboner 1981). Reduced valve adduction in response to repetitive visual stimuli suggests habituation (Fankboner 1981). Compared to visual cues, mechanical and tactile stimuli trigger faster mantle and shell movements (Wilkens 1986). The valve adduction of a 450 mm *T. derasa* following a mechanical stimulus was more rapid (0.37 m s^−1^) than for a shadow response (0.28 m s^−1^).

**Fig. 4:**
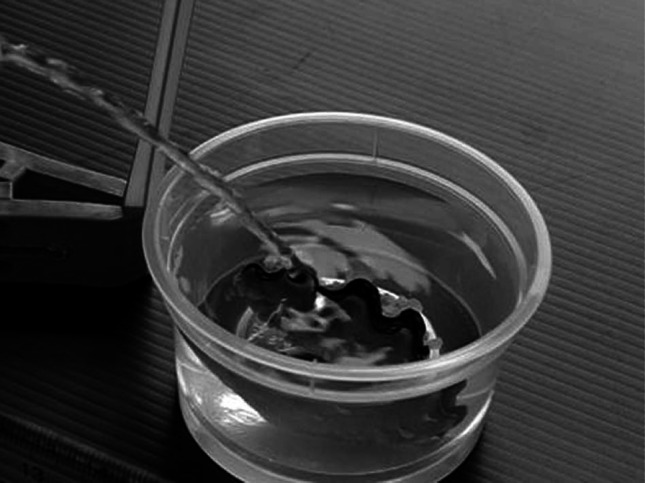
A juvenile *T. squamosa* squirting a jet of water from its exhalant siphon

While filming the behaviour of juvenile *T. squamosa* in the presence of cardboard fish models, Neo (2009) noted both oscillatory and unidirectional squirt patterns and reported an ability to hit these models regardless of their position (i.e. in line with longitudinal axis of the clam, or at 45° to this axis). This suggests that ‘aiming’ with the exhalant siphon, as described in adult *T. maxima* adults (Stasek 1965), also occurs in juvenile clams. Neo and Todd (2011b) analysed stills from video recordings of squirting juvenile *T. squamosa* (for an example see Fig. 4) to calculate the initial velocity, force and pressure exerted by each squirt on an object. They found positive correlations between shell length and the force exerted, but that the pressure produced decreased rapidly with distance from the clam.

### Adults

SCUBA divers often observe that giant clams are able to sense their presence and respond to shadows by closing their valves to varying degrees (Rosewater 1966; Hickman and Gruffydd 1971; Morton 1978). Adults possess numerous (>3,000 for a 900 mm *T. gigas*) pinhole eyes along their mantle margins, constituting the visual mechanism to mediate defensive withdrawal responses (Fankboner 1981). The initial response of *T. maxima* adults to shadows is the overlapping and infolding of the lips of the exhalant cone. Larger species exhibit mantle withdrawal only, while smaller species close their valves in the presence of passing shadows. Flickering shadows cast by small surface waves have no effect. Stasek (1962, 1965) concluded that the shadow reaction is an adaptation to the presence of reef fish such as parrotfish and surgeonfish that gnaw and scrape the coral substratum. Based on in situ observations of adult clams in the presence of roving reef fish, Stasek (1965) described giant clams as being able to perceive moving objects. Even though shadows of the fish did not fall on the clam, the presence of small pomacentrids caused partial and temporary retraction of mantle lobes while larger fish elicited incomplete valve closure.

Giant clams at rest have valves that invariably gape open to maximise light capture by the hypertrophied mantle tissues which house millions of photosynthetic zooxanthellae (Yonge 1936; Hickman and Gruffydd 1971). Stasek (1965) observed no visible response to hammering, scratching on coral or fluttering hand movements below the lateral mantle expansions. Vibrations travelling through the water were thus not the stimulus necessary to elicit mantle retractions typical of shadow responses. However, direct mechanical stimulation of the mantle can elicit valve adduction in adult giant clams, often generating a forceful jet of seawater from the exhalant siphon (McMichael 1974; Morton 1978). Numerous observations of such squirting reflexes have been reported by SCUBA divers and scientists; for example, McMichael (1974, p. 254) noted that it was ‘an occupational hazard when measuring clams to be regularly squirted in the face’.

Stasek (1965) concluded that the aiming-spurting behaviour which he observed in *T. maxima* adults is not of unique adaptative significance. He argued that, while the jet of water produced by valve adduction is sufficiently strong to drive away small fish, it is likely only enough to induce a temporary startle reaction in larger fish. Even so, startling may provide enough time for the clam to retract its exposed mantle lobes into its shell (Wilkens 1984). While it is clear that squirting is a common outcome of the rapid adduction of valves, its function as an anti-predatory behaviour in situ has yet to be fully tested.

## Stress responses

Marine bivalves, including cockles, are known to be susceptible to contaminants and other stressors (Malham et al. 2012). The effects of environmental and anthropogenic factors on giant clams are not well-established, although the ability to sense and respond to such impacts undoubtedly influences their survival (Elfwing et al. 2001). Only a handful of papers have studied giant clam responses to stress (e.g. Svane 1996); these include sediment and sand loading, physical dislodgement, exposure to heavy metals, reduced salinity, and elevated temperatures.

### Larvae

Even though larval stages of invertebrates are generally sensitive to environmental changes, research on stress responses during early giant clam development is very limited. Blidberg (2004) demonstrated that decreased salinity and exposure to low doses of copper are synergistic stress factors for *T. gigas* larvae. Greater numbers of swimming veligers suggested an inhibition or delay in metamorphosis, similar to other invertebrates exposed to heavy metal contaminants (e.g. Lyons et al. 2002). The extension of the pelagic larval stage may act as a biological cue for toxicity and explain the anti-settling behaviour of giant clam larvae near populated coastal areas and river mouths (Blidberg 2004). Neo et al. (2013a) tested the effects of temperature (~22.5 vs. ~29.5 °C) and salinity (27 vs. 30 ‰) in *T. squamosa*. They determined that fertilisation success was significantly greater at the higher temperature, but resulted in almost total mortality at 24 h. They found no significant differences in either embryo or trochophore numbers between the two salinities. A subsequent study by Eckman et al. (2014) noted that *T. squamosa* trochophores exposed to salinities of 9 ‰ (up to 3 h) and veligers exposed to salinities of 12 ‰ (up to 42 h) stopped swimming and sank to the bottom of the test containers. However, when returned to fresh seawater, they resumed their normal swimming activity within an hour. In the same study, late-stage pediveligers or early-stage juveniles (2.3–3.0 mm shell length) responded to zero-salinity distilled water by withdrawing their mantle tissues and siphons and closing their valves. After up to 5 h under these conditions, all specimens recovered within 30 min of being returned to seawater (Eckman et al. 2014).

### Juveniles

Living in association with shallow-water coral reefs, giant clams are often subjected to wave action and wave-induced re-suspension of sediments (Lund-Hansen et al. 1999). Purchon (1955b) was one of the first authors to note that entry of sand and sediments into the mantle cavity of bivalves through the siphonal apertures could potentially cause organ damage. If the guard tentacles at the inhalant aperture fail to stop such unwanted materials, it can be removed from the mantle cavity by ciliated waste canals or by jets of water ejected through the exhalant siphon. Elfwing et al. (2001) identified three behavioural responses of *T. squamosa* juveniles placed in sediment-loaded waters: partial contraction of mantle, full contraction of mantle, and mantle cavity exhalation. The intensity of behavioural activity recorded for sediment treated clams was significantly higher than for control clams. Elfwing et al. (2001) also demonstrated that previous exposure to copper increased the activity of clams then exposed to sediments. To further quantify the behavioural responses to sand deposition in juveniles, we individually exposed 16 *T. squamosa* clams (26–71 mm) to 1-g reef sand deposited from a height of ~50 mm. The responses of the clams were videoed for 1 h. The juveniles removed sand by mantle contractions and ejecting water. The behavioural responses to sand loading were similar to the three described by Elfwing et al. (2001), except that we also observed water being ejected through the inhalant siphon, not just the exhalant. Qualitatively, the clams appeared to be able to remove most of the sand within the hour of video recording. The behavioural responses to silt (Elfwing et al. 2001) and sand (present study) suggest a certain level of resilience to the physical effects of sediment stress.

Righting is a critical biological function in molluscs (e.g. Peck et al. 2004). A common behaviour in bivalves, it involves the active re-alignment to a vertical position after physical displacement (Waller et al. 1999). Giant clams gain a substantial portion of their energy from phototrophy, and an upright posture favours maximum light capture. Filter feeding and locomotion are also best achieved when upright. Without byssus for anchorage to the substrate, free-living species such as *T. gigas* and *H. hippopus* have a higher predisposition to be toppled by wave action or swimming predators (Fankboner 1971). To observe righting behaviour in juvenile *T. squamosa*, we used a wooden rod to repeatedly topple 14 individuals (14–54 mm in shell length). We noted two methods of righting behaviour, each only taking a matter of seconds. Smaller individuals (up to 30 mm) tended to use the foot as an anchor (the foot was seen to repeatedly extend then retract through the pedal gape before attaching to the substrate) and valve adduction to jerk upright. In contrast, larger juveniles (>30 mm) relied on rapid and forceful valve adduction to return to an upright position. This method used a strong jet of water expelled through the exhalant siphon to create a ‘push’ force for righting.

Ecotoxicological research has focused mainly on juveniles (e.g. Blidberg et al. 1999; Elfwing et al. 2002, 2003). Sub-lethal exposure to copper (50 μg l^−1^) and low salinity (20 psu) can significantly lower the ratio between gross photosynthetic production (*P*_g_) and respiration (*R*) values (Elfwing et al. 2001). Copper concentrations much higher than 50 μg l^−1^ have been reported in reef waters (review by Peters et al. 1997). Sub-bleaching temperature levels can affect photosynthetic performance, reducing the energy available for growth and reproduction (Blidberg et al. 2000). When ambient temperature is raised by three degrees over 24 h, *T. gigas* and *T. derasa* juveniles display negative trends in net oxygen production and respiration rates but higher *P*_g_:*R* ratios due to decreased respiration (Blidberg et al. 2000). There is also a difference in sensitivity to heat stress, with photosynthetic efficiency decreasing most in *T. derasa* and metabolic demand increasing most in *H. hippopus* (Blidberg et al. 2000).

### Adults

Within the literature on giant clam adults, there are fragments of information regarding the effects of physical displacement and high temperatures. Only Fankboner (1971) has attempted to explain righting behaviour in adults in relation to shell morphology. While byssate species such as *T. squamosa* and *T. crocea* are able to anchor onto the substrate (Yonge 1936), free-living species may be toppled by strong waves during storm conditions, or roll laterally for a short distance across the reef (Purchon 1977). Yet, they are rarely seen in any position other than upright (Fankboner 1971). In addition, the lack of size stratification with depth suggests that *T. derasa* adults are not adversely affected by wave-induced turbulence (Adams et al. 1988).

So far, two hypotheses have been proposed for how giant clams regain their original position: an ‘automatic righting system’ (Purchon 1977) and a step-wise, self-righting response (Fankboner 1971). Purchon (1977) noted that heavy deposits of nacre occur within the umbonal region. This thickening enhances postural stability by lowering the clams’ centre of gravity, hence the ‘automatic righting system’. Regardless of how the shell is rocked and rolled over by the waves, it will always come to rest with the umbones lowermost given their substantial mass (Hickman and Gruffydd 1971). Fankboner (1971) contested this idea with his observations that clams go through a righting response after toppling. While not ruling out Purchon’s theory of righting as an inherent benefit from modified shell morphology, he noticed that toppled clams in the field (Eniwetok Atoll, Marshall Islands) do not automatically right themselves. Instead, *T. gigas* and *H. hippopus* adults both engaged in a stepwise sequence leading to righting. When toppled over by wave action, the valves remain closed for a period of time, between 1 and 72 h, after which the adductor muscle relaxes and the hinge ligaments spring the valves apart. The displacement of the uppermost heavy umbone to one side acts as a counterweight for self-righting (Fankboner and Reid 1990).

For zooxanthellate reef organisms such as corals, an increase in seawater temperature by a few degrees above the seasonal maximum is likely to induce the dissociation of symbionts (Glynn 1993). This process of bleaching has been recorded several times in giant clams and manifests in the expulsion of *Symbiodinium*, resulting in the loss of colour from mantle tissues (Braley 1986; Gomez and Mingoa-Licuanan 1998; Gomez et al. 2000). Mantle withdrawal beyond the edges of the valves is also common and over prolonged periods of thermal stress precedes death. In some cases, small bubbles form under the epidermal layer of the mantle of a heat-stressed clam, but recovery has been observed within 1 month (Braley 1986). The ability of giant clams to host multiple *Symbiodinium* clades, including clade D that is known to be thermally tolerant (Stat and Gates 2011), may confer some resistance to thermal stress anomalies.

## Why understanding giant clam behaviour is important for their conservation

Giant clam populations throughout their range are disappearing at an alarming rate due to anthropogenic impacts such as overexploitation for food and the aquarium trade, pollution, and habitat loss. Once clam density reaches a point where it becomes unlikely that the gametes of these broadcast spawners can meet and fertilise (the component Allee effect), the ability for a population to self-sustain is compromised (Neo and Todd 2012b). Under such conditions, one of the few conservation options is to intervene and restock to raise densities to levels that increase the probability of natural recruitment. Mariculture of giant clams can produce large numbers of individuals suitable for restocking corals reefs and has contributed to a number of successful restoration efforts (e.g. Soloman Islands, Bell 1999; Philippines, Gomez et al. 2000).

The first step in mariculture is to produce gametes for fertilisation, and intragonadal injection of serotonin has proven to be most effective method in stimulating individuals to release sperm and eggs; however, it also causes some stress to the clams. Even though the exact cues for spawning in the wild have yet to be elucidated, giant clams display diel and lunar periodicities in reproduction and general peak breeding seasons have been established for some species. Induced spawning should therefore be planned around these time frames to take full advantage of their natural reproductive cycles (Ellis 1998). Existing conflicts in short-term monitoring spawning data could be resolved with long-term in situ monitoring of spawning behaviour and fecundity (e.g. Fujikura et al. 2007). Future work could also test for plasticity in spawning—to examine whether it is possible to advance or delay gamete maturation in order to exploit optimal spawning conditions (Tan and Yasin 1998).

Despite the substantial body of research into rearing techniques, ex situ cultured giant clams generally experience high mortality during metamorphosis and growth (e.g. Jameson 1976; Fitt et al. 1984; Ellis 1998). For example, complex switches in the mode of acquiring nutrition as larvae develop into juveniles were highlighted in the section on feeding behaviour. Unsuitable feeding regimes, for pedal feeding at larval stages in particular, are a probable cause for high post-metamorphic mortality rates (Reid et al. 1992). It is essential to provide early pedal-feeding larvae an environment comprising physical properties matching this mode of feeding, especially in terms of substrate rugosity and the size of food particles (Reid and King 1988). Determining species-specific timelines for changing modes of nutrition should similarly be a research priority.

Locomotion in giant clams also has implications for mariculture and restocking programmes. Juvenile giant clams exhibit chemotaxis and natural aggregative behaviour; hence, constant separation of clumped individuals in culture may not be necessary. Any one of the three possible ultimate functions of clumping highlighted by Huang et al. 2007): protection from predators, stabilisation and increased chance of fertilisation, are sufficient reasons to place clams in groups when transplanted onto a reef (Neo and Todd 2012a). Assuming that it is possible to position maricultured clams at high enough densities to encourage natural spawning and fertilisation, it is equally important to ensure that any resultant larvae have the best possible chance of finding a suitable site to settle and grow. The source-sink dynamics of giant clam larval dispersal are central to any restocking programme as nurseries should ideally be located on known source reefs so they can seed sink sites (Neo et al. 2013b). Larval dispersal patterns are usually predicted using hydrodynamic models; however, these require good behavioural data, such as the speed and swimming directionality of pelagic larvae (Neo et al. 2013b) and settlement cues (Neo et al. 2009).

Even if sources and sinks can be determined, there are still many variables that can affect the probability of larvae successfully recruiting. For example, a better understanding of how stressors such as heavy metals, low salinity and increased temperature influence the duration of pelagic stages and settlement behaviour is needed. Sediment, in particular, continues to be a major pollution issue within the ranges of most giant clam species (Todd et al. 2010). Sediment layers on substrates are known to impede invertebrate larval settlement (e.g. Rogers 1990; Te 1992) and are likely to negatively influence giant clam larvae too. Exactly how giant clam larvae behaviourally respond to sediment-covered surfaces, however, has not been established.

Predation plays an important role in shaping bivalve mollusc communities. Visually mediated behaviours such as shadow and sight responses form a major component of the anti-predatory mechanisms employed by juvenile and adult giant clams (Wilkens 1986; Stasek 1965). Visual responses (i.e. sudden retraction of the colourful mantle and valve adduction) are likely to be more important in startling potential predators than the squirting response from either siphon apertures (Neo and Todd 2011b). Nevertheless, prolonged predatory pressures that result in repeated valve closure, and hence interrupted feeding and mantle exposure, will affect giant clam energy budgets and growth rates. Endurance of intense predation pressure and the ability of giant clams to acclimatise/habituate to repeated disturbances have implications for their survival and identification of nursery areas, but have yet to be investigated.

**Fig. 5:**
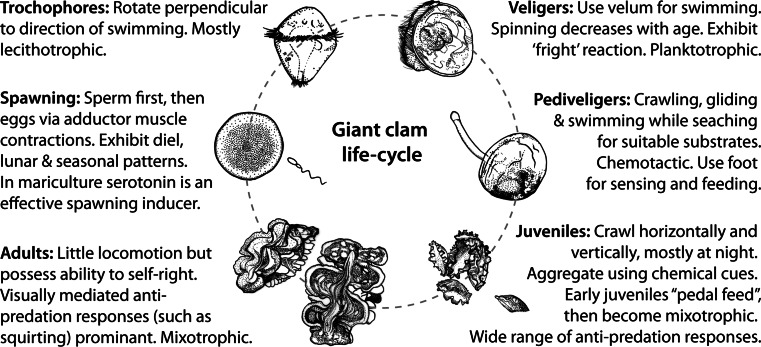
Behaviours associated with different stages of the giant clam’ life cycle

Giant clams are significant ecological components of coral reefs, and their decreasing populations are of concern. While existing mariculture techniques have successfully bred juvenile giant clams in their thousands, future restocking strategies should take into greater consideration their behavioural ecology (summarised in Fig. 5). Behavioural studies are a key part of fish mariculture development (e.g. Salvanes and Braithwaite 2006), and a similar approach of applied and directed research into giant clams can only benefit their conservation and management.

## Conclusions

Giant clam research during the last century has focused largely on the evolution of the clam-zooxanthellae symbiosis and optimising growth rates for mariculture. For an animal that has been relatively well studied, it comes as a surprise that its behaviour has received relatively little scientific attention. Of the 100 or so publications containing behavioural data, approximately a third is related to spawning patterns. No behavioural information has been recorded for *Tridacna mbalavuana*, *T. rosewateri*, *T. squamosina* and *T. noae*. Much room is left for verification and quantification of the anecdotal evidence of behaviours scattered throughout the literature.

Even though the exact cues for spawning in the wild have yet to be elucidated, tridacnines appear to display diel and lunar periodicities in reproduction. General peak breeding seasons for giant clams have been established for some species, but long-term in situ monitoring of spawning behaviour and fecundity is lacking. Determining the critical size of a reproductive unit within a population would be very useful for conservation managers (Braley 1986). Information on the reproduction of giant clams in their natural environment is important for predicting their recovery rate on reefs within depleted populations (Yamaguchi 1977). To date, intragonadal injection of serotonin has proved to be most effective method for spawning induction. This technique can be optimised by timing induction to coincide with the clams’ natural reproductive cycle (Ellis 1998).

Giant clams have considerable mobility, ranging from swimming and gliding as larvae to crawling in juveniles and adults. At the pediveliger and juvenile stages, crawling and propulsion by rapid valve closure are common behaviours during the settlement period. Much of how locomotion patterns and tactic responses are shaped by environmental factors remains unknown. However, juveniles (of *T. squamosa* at least) move more at night than during daytime, exhibit chemotaxis and geotaxis, but they do not move towards light. The clumping behaviour described by Huang et al. (2007) should be taken into consideration when transplanting or restocking giant clams as it may increase survival. Knowledge of pelagic larval movement, such as their speed and swimming directionality, coupled with hydrodynamic modelling, would allow researchers to better predict larval dispersal and recruitment patterns.

Giant clams undergo a series of transitions in feeding strategy as they develop from larvae to juveniles (the same period during which they lose most of their locomotive capabilities) yet these shifts (e.g. to pedal feeding) are generally poorly studied. A collation of a species-specific timeline for changing modes of nutrition and locomotion should be a research priority for conservation and/or commercial mariculture programmes. In mariculture, unsuitable feeding regimes are a probable cause for high post-metamorphic mortality rates. It is essential to provide early juveniles with substrates with physical properties matching this mode of feeding, especially in terms of rugosity and food particle size (Reid and King 1988).

Because of their shell weight and/or byssal attachment, adult giant clams are unable to immediately engage in locomotory escape behaviours upon sensing, or contact with, predators. Nonetheless, they can respond to both shadows as well as tactile stimulation. Visually mediated behaviours such as shadow and sight responses form a major component of the anti-predatory mechanisms employed by both juvenile and adult giant clams (Wilkens 1986). The squirting described by Stasek (1965) and Neo and Todd (2011b) probably has multiple functions including predator deterrence and removal of rejecta from the mantle cavity. How well giant clams can ‘aim’ their squirts remains to be empirically tested. Visual responses, i.e. sudden contraction of the colourful mantle and valve adduction, are likely to be more important in startling potential predators than the squirting response from either siphon aperture. The effects of prolonged predatory pressures and associated valve closure on feeding behaviour and phototrophy in giant clams also have implications for their energy budget and growth rates.

The common misconception that giant clams are sedentary and simple animals stems mainly from a poor appreciation of their sensory systems and how they respond to environmental stressors such as wave action and changes in seawater properties. Distinctive righting behaviours have been described for juveniles and adults, but the effects of repeated physical disturbances, temperature fluctuations and pollution impacts are unknown. As they are expected to depress activity (Elfwing et al. 2001), more work could be done to identify synergistic effects of multiple environmental stressors on giant clam behaviour. Sensitivity thresholds to stress may be indicative of physical vigour (Waller et al. 1999) and could be a basis for selective breeding, especially for clam restocking programmes.

Knowledge on the behaviour of giant clams has applications for conservation efforts and the fine-tuning of mariculture techniques. Comparative data, both quantitative and qualitative, would help to address behavioural and ecological requirements specific to each species (Adams et al. 1988). Understanding the repertoire of giant clam behaviours will also facilitate the prediction of threshold levels for sustainable exploitation as well as recovery rates of depleted clam populations on the Indo-Pacific’s disappearing reefs.
